# Effectiveness and safety of exenatide in Korean patients with type 2 diabetes inadequately controlled with oral hypoglycemic agents: an observational study in a real clinical practice

**DOI:** 10.1186/s12902-017-0220-4

**Published:** 2017-10-25

**Authors:** You-Cheol Hwang, Ari Kim, Euna Jo, Yeoree Yang, Jae-Hyoung Cho, Byung-Wan Lee

**Affiliations:** 1 Division of Endocrinology and Metabolism, Department of Internal Medicine, Kyung Hee University School of Medicine, Kyung Hee University Hospital at Gangdong, Seoul, South Korea; 2 AstraZeneca, Seoul, South Korea; 3 Division of Endocrinology and Metabolism, Department of Internal Medicine, Seoul St. Mary’s Hospital, College of Medicine, The Catholic University of Korea, 222 Banpo-daero, Seoul, Seocho-gu 06591 South Korea; 4 Division of Endocrinology and Metabolism, Department of Internal Medicine, Severance Hospital, Yonsei University College of Medicine, 50-1 Yonsei-Ro, Seoul, Seodaemun-Gu 03722 South Korea

**Keywords:** Exenatide, Type 2 diabetes mellitus, Glucose, Adverse event, GLP-1 analogue

## Abstract

**Background:**

Randomized clinical trials have shown the efficacy and safety of short-acting exenatide in patients with type 2 diabetes mellitus (T2DM). The aim of this observational study was to investigate the effectiveness and safety of exenatide twice a day in Korean patients with T2DM who are suboptimally controlled with oral hypoglycemic agents.

**Methods:**

This study was a post hoc analysis of multi-center (71 centers), prospective, observational, single-arm, post-marketing study of short-acting exenatide 5 to 10 μg twice a day from March 2008 to March 2014 and analyzed those who finished the follow-up over 20 weeks of medication. Changes of hemoglobin A1c (HbA1c), fasting plasma glucose (FPG), and body weight values before and after exenatide treatment were analyzed. Adverse events and adverse drug reactions were estimated in patients who were treated with exenatide at least once and for whom follow-up for safety has been completed.

**Results:**

After 20 weeks treatment with exenatide, mean HbA1c and body weight were significantly reduced from 8.4% to 7.7% and from 83.4 kg to 80.2 kg, respectively (both *p* < 0.001). Subjects with higher baseline glucose and HbA1c levels showed an independent association with a greater reduction in glucose level. In addition, short duration of diabetes less than 5 years was an independent predictor for the improvement in glucose level. The majority of study subjects showed a reduction in both body weight and glucose level (63.3%) after exenatide treatment. In terms of safety profile, exenatide treatment was generally well-tolerated and the incidence of severe adverse event was rare (0.8%). The gastrointestinal side effects were most common and hypoglycemia was reported in 1.7% of subjects.

**Conclusion:**

In real clinical practice, 20 weeks treatment with short-acting exenatide was well tolerated and showed a significant body weight and glucose reduction in Korean patients with T2D who are suboptimally controlled with oral hypoglycemic agents.

**Trial registration:**

ClinicalTirals.gov, number NCT02090673, registered 14 February 2008.

**Electronic supplementary material:**

The online version of this article (10.1186/s12902-017-0220-4) contains supplementary material, which is available to authorized users.

## Background

The prevalence of obesity and type 2 diabetes mellitus (T2DM) is now increasing rapidly in Korea and Asian countries [1–3]. However, glycemic control is not satisfactory and the proportions of patients with reaching target hemoglobin A1c (HbA1c) of <6.5% and <7.0% were 27% and 45.6%, respectively in Korea [4]. In addition, different side effects related to oral hypoglycemic agents including weight gain and hypoglycemia [5] can cause a nonadherence to medications and poor glycemic control [6]. Therefore, there is still unmet need for another class of hypoglycemic agent has both good efficacy and safety.

Exenatide is an incretin mimetic that activates glucagon-like-peptide-1 (GLP-1) receptors. It mainly decreases postprandial plasma glucose level by stimulating insulin secretion, suppressing glucagon secretion and delaying gastric emptying in a glucose-dependent manner [7, 8]. Numerous clinical studies in Western countries have shown the glucose-lowering efficacy of exenatide monotherapy [9] and in combination with other oral hypoglycemic agents including sulfonylurea, metformin, and thiazolidinediones [10–13].

However, the pathophysiology of T2DM could be different according to ethnicity and it was reported that *β*-cell dysfunction is a main pathogenic mechanism for the development of T2DM in Asian population but, a lesser degree of insulin resistance compared with Caucasian [1, 14]. In addition, glucose-lowering effect of GLP-1 analogues was reported to be greater in Asian-dominant studies than in non-Asian-dominant studies [15]. Furthermore, it has been well known that results from real clinical setting are different from those from randomized clinical trial (RCT) and thus, observational studies are also necessary to evaluate the effectiveness of health care [16].

Therefore, the aim of this study was to investigate the effects of exenatide twice a day injection in a real clinical practice on blood glucose level, body weight, and safety profiles in obese Korean patients with T2DM who are suboptimally controlled with oral hypoglycemic agents.

## Methods

### Study design

This is a post hoc analysis of multi-center (71 centers), prospective, observational, single-arm, post-marketing surveillance (PMS) study to evaluate the safety and effectiveness of short-acting exenatide (Byetta®), conducted from March 2008 to March 2014, in patients with T2DM (ClinicalTrials.gov, number NCT02090673). The investigators’ decisions regarding the proper treatment and care of the patient were made in the course of the normal clinical practice; without blinding or randomization to particular comparator arms or therapies. In this study, patients were enrolled for the collection of their data on observations made during normal clinical practice such as serum HbA1c, fasting plasma glucose (FPG), body weight, medical history, and adverse events.

### Study populations and treatments

The patient population for this study consisted of Korean patients who were at least 18 years old, diagnosed with T2DM, and were treated with short-acting exenatide in an ambulatory care setting according to the approved label. Patients were excluded from the study for any of the following reasons; simultaneously participating in a different study that includes a treatment intervention and/or an investigational drug, being pregnant or having intentions to be pregnant within the duration of the study, and being contraindicated for short-acting exenatide. Investigators have ensured that patients, or, in those situations where consent could not be given by patients, their legally acceptable representatives, are clearly and fully informed about the purpose of the study, potential risks, the patient’s rights and responsibilities when participating in this study.

All patients were initially injected using 5 μg of short-acting exenatide twice daily. After 1 month of injection 5 *μ*g, the exenatide dosage was intended to increase to 10 *μ*g twice a day under the decision of the physician. The previous antidiabetic medications were maintained during the periods of exenatide treatment. Life style modification therapy and education for not only diet but also exercise were combined in parallel with medications.

### Effectiveness assessment

**Fig. 1:**
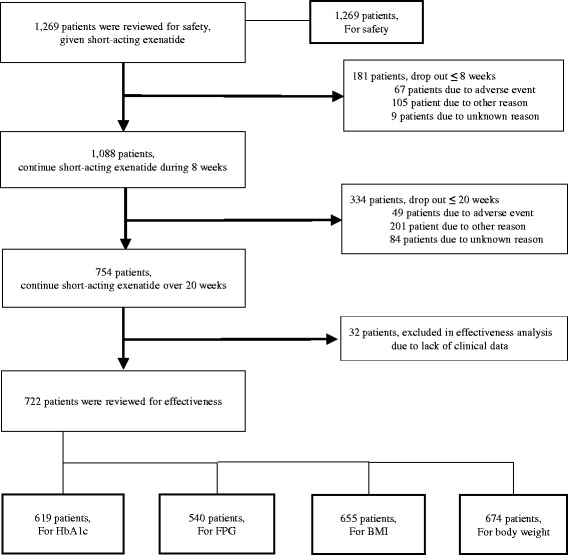
Study flow diagram demonstrating analysis population

Based on electronic database of the study with high confidentiality, the patients were rearranged for the purpose of reanalysis settings (Fig. 1). The variables for the effectiveness of short-acting exenatide after 20 weeks of treatment were changes in HbA1c, FPG, body mass index (BMI), and body weight. Total 1269 patients who received more than one-time injection of exenatide were reviewed. Of these, 181 and 334 patients dropped out within 8 and 20 weeks, respectively. Thus, 754 patients continued short-acting exenatide over 20 weeks and were reviewed for safety analysis. With additional exclusion of 32 patients due to lack of clinical data, a final number of 722 patients were reviewed for effectiveness analysis. Among them, 619 patients who measured HbA1c level both at baseline and 20-week endpoint were analyzed for HbA1c. In the same way, 540, 655, and 674 patients were analyzed for FPG, BMI, and body weight, respectively. Patients were also defined by 2-dimensional quadrant allocation according to the absolute change in HbA1c and body weight in the same picture.

Reanalysis for the effectiveness was conducted using stratified categorization with the responsiveness for the short-acting exenatide. In the first stratum by median value of HbA1c reduction, patients were divided into two groups with 0.6% HbA1c reduction. The second stratum used the absolute value as target HbA1c as 7.5%. As known in various randomized controlled trials, mean HbA1c at the end of treatment, the primary endpoint in the majority of studies, was decreased by 0.79 to 1.75% (the median value across these large studies was 1.11%) [17]. The baseline HbA1c of our study was 8.4% and we assumed responder as patients who would reach HbA1c level at 7.5% after 20 weeks of exenatide treatment. On the other hands, the third stratum was applied by the mean change percentage of HbA1c from baseline which was calculated as 9.8% as percentage. The mean HbA1c level was 8.40% before treatment and 7.56% after exenatide over 20 weeks. The absolute difference was 0.84%, which was re-calculated as 9.8% as percentage compared to basal mean HbA1c level.

### Safety assessment

Patients who were participating the study could reject or withdraw the continuation of treatment or the study with the reason of follow-up lost, adverse events, patient’s decision, or physician’s decision. If a patient discontinued the treatment during the study, the patients were contacted to collect all adverse events information and reasons why they want to stop the treatment. All relevant information was captured by data form. Patients who were discontinued from study were included to safety population if they had taken at least one dose of exenatide. We calculated the discontinuation rate due to any adverse events. Adverse events and serious adverse events which were recorded in electronic case report form were recoded and reanalyzed according to the current routine classification. For the serious adverse events, we reviewed the patient record and revealed the diagnosis of them.

### Statistical analysis

All statistical analysis was performed with SAS (version 9.4, SAS Institute, Cary, NC, USA) for the numerics, and NCSS (version 11, NCSS company, Kaysville, UT, USA) for the graphics. For effectiveness analysis, missing values was imputed using the last observation carried forward methods. Baseline characteristics were shown in descriptive statistics such as mean and standard deviation for continuous data, or frequency and percentage for categorical data. Safety analysis was also summarized descriptively.

Clinical factors which could affect the HbA1c reduction between responder and non-responder groups were analyzed using chi-square test in 3 different strata. We performed multivariate logistic regression test to adjust the potential variables for the HbA1c reduction in each stratum. To confirm the correlation between changes of HbA1c and body weight, scattered graph was rendered and correlation coefficient was calculated. If *p*-value is less than 0.05, it was considered statistically significant.

## Results

### Baseline clinical and laboratory characteristics of participants

Of the total 1269 patients who received more than one-time injection of exenatide twice a day, data retrieved from 754 patients who completed 20 weeks treatment with exenatide were analyzed for the safety profiles (safety analysis set). Among the 754 participants, 619 patients who measured HbA1c level both at baseline and 20-week endpoint were analyzed for the effectiveness (effectiveness analysis set).

Based on the safety analysis set (*n* = 754), the mean age and BMI of study subjects were 48.8 years and 31.2 kg/m^2^, respectively. 34.0% of the subjects were men. Approximately one third of the study subjects had a family history of diabetes and duration of diabetes was 7.7 years. At baseline, mean HbA1c and FPG level were 8.4% and 164.3 mg/dL, respectively (Table 1).

**Table 1:** Baseline characteristics of the study subjects

Baseline demographics	Patients who completed at least 20 weeks of short-acting exenatide (*n* = 754)
Age (years)	48.76 ± 11.44
Sex	
Male	256 (33.95)
Female	498 (66.05)
BMI (kg/m^2^)	31.15 ± 4.68
Baseline HbA1c (%)	8.40 ± 1.74
Baseline FPG (mg/dL)	164.26 ± 62.10
Daily dose (*μ*g)	15.96 ± 3.46
Diabetes family history	
No	491 (65.12)
Yes	263 (34.88)
Duration of diabetes (months)	92.66 ± 79.42
Concurrent disease	
No	115 (15.25)
Yes	639 (84.75)
Antidiabetic medication	
0 or 1 medication	260 (34.48)
2 or more medications	494 (65.52)
Kind of antidiabetic medications	
Metformin	672 (89.12)
Sulfonylureas	494 (65.52)
Thiazolidinediones	17 (2.25)
Other concomitant medication	
No	80 (10.61)
Yes	674 (89.39)

Values are presented as mean ± standard deviation of the mean or the number with percentage

*Abbreviations: BMI* Body Mass Index, *HbA1c* Hemoglobin A1c, Fasting plasma glucose

### Clinical and laboratory variables affecting glucose lowering effectiveness

In the current study, mean dosage of exenatide was 16.0 μg/day. A significant reduction in FPG (15.12 mg/dL) and HbA1c (−0.75%) levels from baseline were observed over 20-week of exenatide treatment. To determine glucose-lowering effect of exenatide, we used three different criteria to define responder to exenatide treatment. The characteristics of responders by three definitions as stratum are shown in Table 2. Using a responsiveness based on absolute value of HbA1c reduction ≥0.6%, subjects with optimal response to exenatide had significantly higher baseline FPG and HbA1c levels than those with suboptimal response to exenatide treatment. In addition, men and subjects with short duration of diabetes less than 5 years were more likely to improve in glucose level with exenatide treatment with significance. However, age and BMI did not affect glucose-lowering effectiveness of exenatide treatment. Using a responsiveness based on achievement in HbA1c ≤ 7.5% at 20-week endpoint, subjects who reached the target HbA1c had statistically lower baseline FPG and HbA1c levels. The subjects with short duration of diabetes showed significantly better glucose control. Using a responsiveness based on achievement in ≥9.8% of baseline HbA1c, subjects with optimal response to exenatide had significantly higher baseline FPG and HbA1c levels. Similar to analysis stratum 1, subjects with short duration of T2DM were more likely to improve in glucose level with exenatide treatment with significance. Before the initiation of exenatide, patients who had been taking 2 oral antidiabetics showed relatively higher proportion in responder in stratum classified by the changes from baseline. However, in the analysis of target HbA1c as 7.5%, patients with 0 or 1 antidiabetics demonstrated better achievement proportion. To determine which clinical and laboratory variables were independently associated with glucose-lowering effectiveness of exenatide, multivariate logistic regression analysis was used. As a result, higher baseline FPG and HbA1c levels were independently associated with greater reduction in glucose level after exenatide treatment. In contrast, subjects with longer duration of diabetes more than 5 years showed an inverse association with the improvement in glucose control (Table 3).

**Table 2:** Clinical factors which may affect HbA1c reduction after treatment with short-acting exenatide over 20 weeks

	HbA1c reduction ≥0.6%			HbA1c <7.5%			HbA1c reduction ≥9.8% from baseline		
	Yes (*n* = 316)	No (*n* = 303)	*P*	Yes (*n* = 346)	No (*n* = 273)	*P*	Yes (*n* = 263)	No (*n* = 356)	*P*
Age (years)									
≥ 50	24.4 (151)	24.9 (154)	0.45	27.1 (168)	22.1 (137)	0.69	20.0 (124)	29.2 (181)	0.36
< 50	26.7 (165)	24.1 (149)		28.8 (178)	22.0 (136)		22.5 (139)	28.3 (175)	
Sex (%)									
Male	19.9 (123)	13.6 (84)	0.003	19.7 (122)	13.7 (85)	0.28	17.3 (107)	16.2 (100)	0.001
Female	31.2 (193)	35.4 (219)		36.2 (224)	30.4 (188)		25.2 (156)	41.4 (256)	
BMI (kg/m^2^)									
≥ 30	31.6 (184)	27.2 (158)	0.13	32.8 (191)	26.0 (151)	0.69	25.8 (150)	33.0 (192)	0.41
< 30	19.6 (114)	21.7 (126)		23.7 (138)	17.5 (102)		16.7 (97)	24.6 (143)	
HbA1c (%)									
≥ 8.5	30.7 (190)	13.6 (84)	<0.001	12.1 (75)	32.2 (199)	<0.001	25.5 (158)	18.7 (116)	<0.001
< 8.5	20.4 (126)	35.4 (219)		43.8 (271)	12.0 (74)		17.0 (105)	38.8 (240)	
FPG (mg/dL)									
≥ 160	27.9 (137)	15.0 (74)	<0.001	17.1 (84)	25.8 (127)	<0.001	23.0 (113)	19.9 (98)	<0.001
< 160	23.6 (116)	33.5 (165)		41.1 (202)	16.1 (79)		19.7 (97)	37.4 (184)	
Daily dose of exenatide (μg/day)									
≥ 15	36.5 (226)	33.9 (210)	0.55	39.6 (245)	13.3 (82)	0.82	31.3 (194)	39.1 (242)	0.12
< 15	14.5 (90)	15.0 (93)		16.3 (101)	30.9 (191)		11.2 (69)	18.4 (114)	
Family history of diabetes (%)									
No	28.4 (135)	24.2 (115)	0.96	29.1 (138)	23.6 (112)	0.86	23.6 (112)	29.1 (138)	0.68
Yes	25.5 (121)	21.9 (104)		26.5 (126)	20.8 (99)		22.1 (105)	25.3 (120)	
Duration of diabetes (years)									
≥ 5	29.4 (158)	32.0 (172)	0.040	26.3 (141)	35.2 (189)	<0.001	23.8 (128)	37.6 (202)	0.023
< 5	22.0 (118)	16.6 (89)		26.8 (144)	11.7 (63)		18.8 (101)	19.7 (106)	
Concurrent disease (%)									
No	6.8 (42)	6.5 (40)	0.97	7.9 (49)	5.3 (33)	0.45	5.8 (36)	7.4 (46)	0.78
Yes	44.3 (274)	42.5 (263)		48.0 (297)	38.8 (240)		36.7 (227)	50.1 (310)	
Number of previous antidiabetic medication (%)									
0 or 1	15.5 (96)	19.1 (118)	0.025	24.2 (150)	10.3 (64)	<0.001	12.8 (79)	21.8 (135)	0.042
2	35.5 (220)	29.9 (185)		31.7 (196)	33.8 (209)		29.7 (184)	35.7 (221)	
Other concomitant medication (%)									
No	3.7 (23)	5.2 (32)	0.15	5.3 (33)	3.6 (22)	0.52	3.4 (21)	5.5 (34)	0.50
Yes	47.3 (293)	43.8 (271)		50.6 (313)	40.6 (251)		39.1 (242)	52.0 (322)	

*Abbreviations: BMI* Body Mass Index, *HbA1c* Hemoglobin A1c,, Fasting plasma glucose

Chi-square test

Data are expressed as percentage (number)

Body mass index, fasting plasma glucose, family history of diabetes, and duration of diabetes were determined in 582, 492, 475, and 537 subjects, respectively

**Table 3:** Adjusted potential variables for the prediction of the HbA1c responses according to the stratified classification

	HbA1c reduction ≥0.6%		HbA1c <7.5%		HbA1c reduction ≥9.8% from baseline	
	OR (95% CI)	*P*	OR (95% CI)	*P*	OR (95% CI)	*P*
Male sex	1.47 (0.94–2.29)	0.09	Not determined		1.40 (0.90–2.15)	0.13
HbA1c ≥8.5%	3.13 (1.95–5.04)	<0.001	0.13 (0.08–0.22)	<0.001	2.36 (1.48–3.77)	<0.001
FPG ≥160 mg/dL	1.60 (1.01–2.54)	0.047	0.58 (0.35–0.97)	0.037	1.47 (0.93–2.34)	0.10
Duration of diabetes ≥5 years	0.997 (0.995–1.000)	0.053	0.994 (0.991–0.997)	<0.001	0.997 (0.994–1.000)	0.043
Number of previous antidiabetic medication (2 vs. 0 or 1)	1.07 (0.67–1.71)	0.79	0.91 (0.52–1.58)	0.74	1.28 (0.80–2.05)	0.31

*Abbreviations: OR* odd ratio, *HbA1c* Hemoglobin A1c, *FPG* Fasting plasma glucose

Multivariate logistic regression test; References of dependent variables in each stratum, Group 2, Group B, and group II, respectively

### Efficacy of Exenatide for HbA1c level and weight changes

**Fig. 2:**
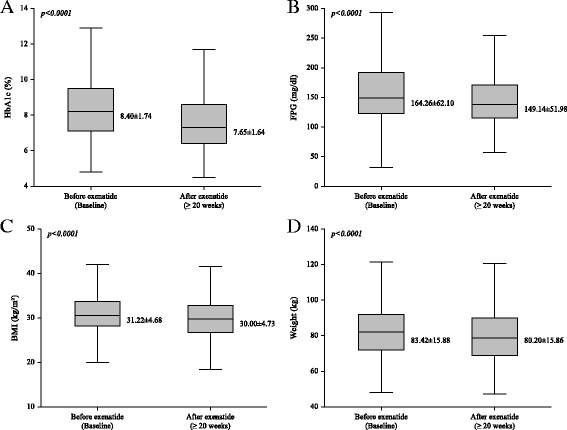
Changes of clinical findings from before to after short-acting exenatide over 20 weeks. (A) Hemoglobin A1c (HbA1c) level (*n* = 619), (B) Fasting plasma glucose (FPG) level (*n* = 54), (C) Body mass index (BMI) (*n* = 655), (D) Body weight (*n* = 674)

**Fig. 3:**
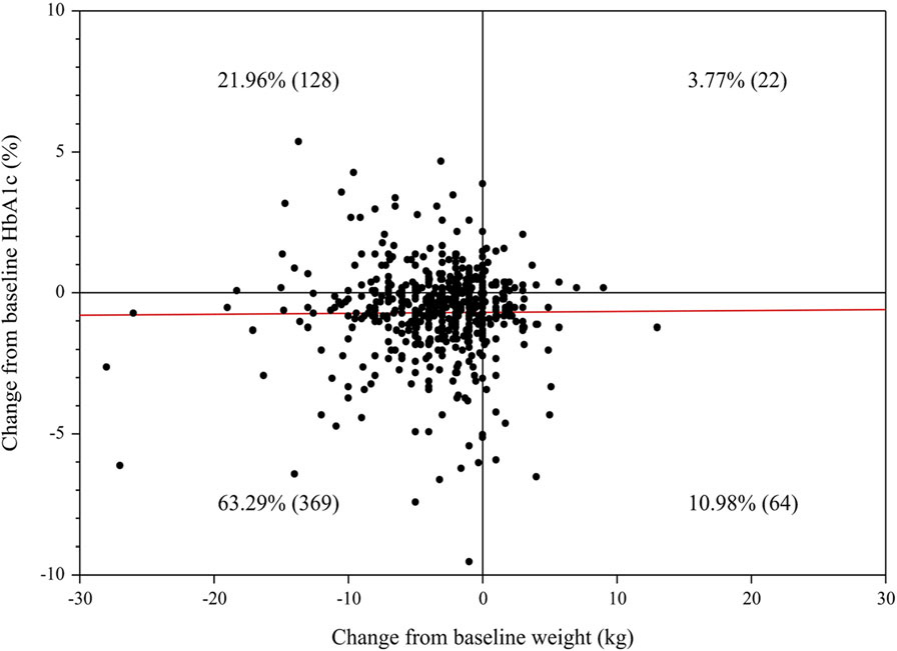
Distribution of patients for the relation between Hemoglobin A1c (HbA1c) and body weight before and after treatment with short-acting exenatide over 20 weeks. Red line represents regression for HbA1c according to body weight change, which shows the correlation coefficient as 0.00357 closed to 0. It means that there is rare correlation between changes HbA1c and body weight

We next determined the weight change with exenatide treatment. After 20 weeks treatment with exenatide twice a day, body weight was significantly reduced from 83.4 kg to 80.2 kg (*p* < 0.001, Fig. 2). The majority of study participants showed a reduction in both body weight and glucose level after exenatide treatment (63.3%) whereas, only 3.8% of subjects did not respond to exenatide treatment in both body weight and glucose aspects. Interestingly, 22.0% of subjects showed a weight reduction but, aggravation in glucose control and 11.0% of subjects showed an improvement in glucose control despite of weight gain (Fig. 3).

### Tolerability and adverse events of Exenatide

Regarding the tolerability of exenatide, 181 and 334 subjects among total 1269 patients discontinued the injection of exenatide twice a day within 8-week and between 8 and 20 weeks of study period. Of 1269 patients, 116 patients stopped the injection of exenatide for adverse events. The final drop-out rate of short-acting exenatide just due to the adverse events was 9.14%. Within 8 weeks of medication, discontinuation rate due to adverse events was 5.3% (67 patients out of 1269 patients). Consecutively, the discontinuation rate due to adverse events between 8 and 20 weeks of medication showed 4.5% (49 patients out of 1088 patients) (Fig. 1).

Based on the safety analysis set (*n* = 754) who completed 20 weeks treatment with exenatide, severe adverse event was very rare (0.8%) but 173 cases (22.9%) of adverse events were reported. The gastrointestinal side effects, mostly nausea, were the most common side effect and hypoglycemia was reported in 1.7% of subjects (Table 4). When it comes to serious adverse events, 6 cases were cholecystitis, hyperglycemia, rotator cuff syndrome, tendon disorder, diabetic skin ulcer, and cellulitis.

**Table 4:** Summary of adverse events for the treatment with short-acting exenatide

Adverse events	Number of patients (n = 754)	Number of events
Any adverse events	173 (22.94%)	246
Serious adverse events	6 (0.80%)	6
Gastro-intestinal adverse events	116 (15.38%)	154
Nausea	93 (12.33)	100
Vomiting	19 (2.52%)	19
Dyspepsia	8 (1.06%)	8
Constipation	7 (0.93%)	7
Diarrhoea	5 (0.66%)	5
Others	13 (1.72%)	15
Metabolic and endocrine adverse events	22 (2.92%)	23
Hypoglycaemia	13 (1.72%)	14
Others	9 (1.19%)	9
Neuropsychologic adverse events	25 (3.32%)	29
Dizziness	7 (0.93%)	7
Headache	6 (0.80%)	6
Anorexia	5 (0.66%)	5
Others	9 (1.19%)	11
Cardiovascular adverse events	5 (0.66%)	5
Respiratory system adverse events	3 (0.40%)	3
Urinary system adverse events	1 (0.13%)	1
Musculo-skeletal or generalized adverse events	16 (1.21%)	18
Others	11 (1.46%)	13

Data are expressed as percentage (number)

## Discussion

To overcome many shortcomings of the classic anti-diabetic drugs (ADDs) such as hypoglycemia and weight gain etc., incretin-based drugs including GLP-1 analogues and dipeptidyl peptidase-4 (DPP4) inhibitors have been progressively developed and been regarded as a recommended drug for glycemic control in patients with T2DM [8]. Furthermore, by overcoming drug delivery system, long-acting GLP-1 s are now gaining popularity as innovative drug in managing the subjects with T2DM. Because of plentiful accumulated reports on exenatide, the first-in-class drug of GLP-1 analogue, the debate on clinical relevance of exenatide might be little. However, the clinical relevance on effectiveness and safety are reported mainly under the designed trials with the statistical expectations. We hypothesized that reluctance might remain to generalize the results of RCTs retrieved from a specific segment of the population to broader population or real practice [16, 18], and that it might be more important to both patients and physicians in managing the diabetes after identifying the results of RCTs with those of observation studies (OSs) retrieved from routine care. Based on these hypotheses, we aimed this study for investigating clinical effectiveness and safety of exenatide twice a day in real-life setting.

From this prospective, 20-week observational, phase 4 PMS study with short-acting exenatide twice a day in Korean patients with T2DM, we demonstrated 3 main findings. First, exenatide twice a day significantly induced HbA1c and weight reduction in real clinical practice. Second, higher baseline glucose level and short duration of diabetes were independent predictor for good glycemic control after exenatide treatment. Third, adverse events of exenatide in real-life setting are similar to those of RCTs [19].

With respect to study design, this analysis retrieved from the prospective, observational, PMS study. RCTs and OSs have both benefits and drawbacks reciprocally, which should be interpreted with balanced stance. Briefly, the inclusion and exclusion criteria of RCTs tend to be defined narrowly to conduct either the regulatory requirements in drug trials or the specific aims targeting selected population. The characteristics of patients who participate in the clinical trials are to be more concerned on their health status and care by health providers, and compliant with the drugs. In these regards, they might provide limited usefulness on the effectiveness and safety of a drug to generalize to the real world practice. In contrast, the design of PMS OSs in everyday practice setting is able to cover a broader population without stringent inclusion/exclusion criteria, and characteristics of patients. Furthermore, it also provides valuable information on the physician’s preference and ideas [20]. These unmet need of RCTs but complemented by OSs as part of routine care might be important to both patients and health providers for treatment decisions.

With respect to the glycemic effectiveness of exenatide twice a day in real-life setting, exenatide improved glycemic control through up-regulating β-cells and down-regulating α-cells residing in pancreatic islets. In recent meta-analysis, short-acting exenatide twice a day was effective in reducing HbA1c (− 0.61% for 5 μg, and – 0.83% for 10 μg) compared with placebo in patients with T2DM [21]. Furthermore, Kim et al. reported that GLP-1 analogues including exenatide, and liraglutide lowered HbA1c more in Asian-dominant studies than in non-Asian-dominant studies [15]. In this OS, there was significant reduction in both FPG (−15.12 mg/dL), and HbA1c (−0.75%) from baseline. Regarding the responders to ADDs, the conventional definitions of responders might be one of the two analyses, the absolute HbA1c change from baseline after treatment, or achievement in a target HbA1c level [22]. Based on this report [22], we analyzed the glycemic effectiveness or responder in three ways; achievement in 1) absolute value of HbA1c reduction ≥0.6%, 2) HbA1c ≤ 7.5%, and 3) ≥ 9.8%, of baseline HbA1c. Using effectiveness or responder definitions based on absolute reduction ≥0.6% HbA1c, achievement in HbA1c ≤ 7.5%, and ≥9.8%, of baseline HbA1c reduction, the percentage rate in responders were 51.1%, 55.9% and 42.5%, respectively. Regardless of definitions of responder, duration of diabetes, baseline FPG and HbA1c, and numbers of ADDs were significantly different between the responders and non-responders. However, the rest two variables are different according to the definitions of responder. Regarding clinical characteristics predicting in responsiveness of exenatide, shorter duration of diabetes was the only predictive variable for responders in glucose control. Those who had higher baseline HbA1c, a critical variable of potential confounder, showed the more reduction in absolute HbA1c and percentage of baseline HbA1c, but the lower achievement in target HbA1c. Previous reports also showed the consistent results that the higher baseline HbA1c was related to the more HbA1c reduction but less probability of achievement in glycemic goal [22–24]. Regarding glycemic effectiveness in response to weight change, treatment with exenatide twice a day significantly reduced - 3.22 Kg body weight. Of the participants who completed the study, 63.3% (*n* = 369) experienced the both benefits of GLP-1 analogues on HbA1c reduction and weight loss. Those who got none benefits of exenatide were just 3.77%. In the study, BMI did not affect glucose-lowering effect of exenatide. In one study which was aiming to investigate metabolic outcomes in patients with type 2 diabetes treated with exenatide, the response in HbA1c reduction was independent of baseline weight, and BMI [25]. Also, most subjects enrolled in this PMS study showed high BMI due to Korean insurance policy during the study period. This may have influenced the outcome of the study.

With respect to the adverse events of exenatide, of 181 participants who discontinued exenatide injection, 37% ones failed to continue on exenatide within 8 weeks. Of 334 participants who discontinued exenatide injection between 8 weeks and 20 weeks, 14.7% of subjects discontinued on exenatide injection. Taken together, of 1269 subjects retrieved from safety analysis set, 161 (9.1%) reported to stop the exenatide injection. Those who were analyzed for effectiveness analysis set (*n* = 754) at 20 weeks in this PMS OS, 22.9% subjects reported any adverse events. Based on this finding, caution should be paid to the patients’ tolerability.

The strengths of this study are the large sample number and the real-world clinical practice setting with patients of only Asian ethnic background. It, however, also has important weaknesses. This study did not provide the laboratory data to evaluate the pancreatic *β*-cell functions and peripheral insulin sensitivity which are the key pathophysiologic factors in the development and progress of T2DM. The clinical implications of duration of diabetes, baseline FPG and HbA1c, and numbers of ADDs which were different between the responders and non-responders might be explained in these assessments. Second, in the balanced stance of both studies design of RCTs and OS, this study might remain observer and selection bias which are reflected by not scientific milieu but socio-economic one. Underreporting also occurred especially in drug adverse events. A final major limitation is the drop out data from missing data and adherence to physicians’ orders.

## Conclusions

In conclusion, the results of the study of real-life clinical practice in Korea demonstrate that, in Korean subjects with T2DM, exenatide twice a day resulted significant reduction in glycemic control and relative tolerance in Korean patients with T2DM in real-world clinical setting.

## Additional file

Additional file 1: Table S1. The institutions and ethic committee’s reference number of this study. (DOCX 23 kb)

## Abbreviations

ADD: Anti-diabetic drug
BMI: Body mass index
Dipeptidyl peptidase-4: DPP4
FPG: Fasting plasma glucose
GLP-1: Glucagon-like-peptide-1
HbA1c: Hemoglobin A1c
OS: Observation study
PMS: Post-marketing surveillance
RCT: Randomized clinical trial
T2DM: Type 2 diabetes mellitus
