# Supplementary material for: Effectiveness and safety of exenatide in Korean patients with type 2 diabetes inadequately controlled with oral hypoglycemic agents: an observational study in a real clinical practice
Source: BMC Endocr Disord. 2017 Oct 25;17:68. doi: 10.1186/s12902-017-0220-4 (PMC5655957; doi:10.1186/s12902-017-0220-4)
Supplement: Additional file 1: Table S1. — The institutions and ethic committee’s reference number of this study. (DOCX 23 kb) [file 12902_2017_220_MOESM1_ESM.docx]

**Table S1. The institutions and ethic committee’s reference number of this study**

| **Institutions** | **Ethic committee’s reference number** |
| --- | --- |
| Gachon University Gil Medical Center | GIRBA2626 |
| The Catholic University of Korea St. Vincent’s Hospital | VC11MSMP0143 |
| Kangnam Sacred Heart Hospital | 2013-10-81 |
| Gangnam Severance Hospital | 3-2010-0070 |
| Gangdong Kyung Hee University Medical Center | KHNHC PMS2010-036,  KHNHC PMS2010-037,  KHNHC PMS2010-038 |
| Kangdong Sacred Heart Hospital | 11-035 |
| Kangbuk Samsung Hospital | KBC11073 |
| Gangseo Miz Medi Hospital | 1202-4 |
| Konkuk University Medical Center | KUH1010233 |
| Konyang University Hospital | 13-45 |
| Gyeongsang National University Hospital | GNUHIRB-2011-030, GNUHIRB-2011-029 |
| Kyung Hee University Medical Center | 0916-07 |
| Keimyung University Dongsan Medical Center | 10-80, |
| Keimyung University Dongsan Medical Center | 10-183, 10-182 |
| Keimyung University Dongsan Medical Center | 11-81, 11-82 |
| Korea University Anam Hospital | AN12050-001 |
| Korea University Ansan Hospital | AS09066-001 |
| Daegu Catholic University Medical Center | CR-11-058 |
| Daejeon Sun Hospital | DSH-약-12-03 |
| Daejeon Eulji Medical Center | 12-049 |
| Bundang Jesaeng Hospital | IME 11-01 |
| Dongkang Medical Center | 2011-03-02 |
| Dongguk University Ilsan Hospital | 2009-1-48 |
| Dongguk University Gyeongju Hospital | 11-20 |
| Maryknoll Medical Center | MMC/2012/04/16-4(142) |
| Myongji Hospital | 10-047 |
| Borame Medical Center | 20110315/06-2011-35/106 |
| Borame Medical Center | 20110701/06-2011-125/109 |
| Pusan National University Hospital | H-1008-001 |
| The Catholic University of Korea Bucheon St. Mary’s Hospital | HC10MSMP0050, HC10MSMP0052, HC10MSMP0054 |
| Bucheon Sejong Hospital | 1136 |
| Soon Chun Hayng University Hospital Bucheon | PMS2011-23 |
| Seoul National University Bundang Hospital | B-0903-072-201 |
| Bundang Cha Medical Center | BD2012-071D |
| Samsung Seoul Medical Center | PMS2009-006 |
| Samsung Seoul Medical Center | SMC PMS2011-016 |
| Samsung Seoul Medical Center | PMS2011-029 |
| Seoul National University Hospital | H-1012-073-344 |
| The Catholic University of Korea Seoul St. Mary’s Hospital | 서275-9-002 |
| The Catholic University of Korea Seoul St. Mary’s Hospital | KC11MSMP0047 |
| Seoul Asan Medical Center | 2010-0146, 2010-0147 |
| Severance Hospital | 4-2008-0635 |
| Severance Hospital | 4-2009-0050 |
| Severance Hospital | 4-2011-0032 |
| Severance Hospital | 4-2011-0480 |
| Soon Chun Hayng University Hospital Cheonan | 2011-101 |
| Soon Chun Hayng University Medical Center | 2011-65 |
| Ajou University Hospital | AJIRB-PMS-PMS-10-296 |
| Ajou University Hospital | AJIRB-PMS-PMS-10-295 |
| Ajou University Hospital | AJIRB-PMS-PMS-11-059 |
| The Catholic University of Korea Yeouido St. Mary’s Hospital | SC11MSMP0018 |
| Yeungnam University Medical Center | PCR-11-66 |
| Ulsan University Hospital | 09-101 |
| Wongkwang University Hospital | 의임상 7303-534 |
| Wongkwang University Sanbon Hospital | 의산본 7302-201004 |
| Inje University Ilsan Paik Hospital | IB-3-1112-053 |
| The Catholic University of Korea Incheon St. Mary’s Hospital | OC10MSMP0043 |
| Cheil Medical Center | CGH-IRB-2009-36 |
| Jeju National University Hostpial | IRB 2009-44 |
| Cheongju St. Mary's hospital | IRB-50 |
| Chungnam National University Hospital | PMS2011-009-001,  PMS2011-010-001 |
| Chungnam National University Hospital | CNUH PMS2011-008 |
| Korean Cancer Center Hospital | K-1206-002-013 |
| Kangnam Sacred Heart Hospital | 2009-04-20 |
| Hallym University Sacred Heart Hospital | 2010-S059 |
| Hanyang University Medical Center | HYUH IRB 2011-C-04, HYUH IRB 2011-C-05, HYUH IRB 2011-C-06 |
| Hanil General Hospital | 2012-009 |
| Inje University Haeundae Paik Hospital | 2010-062 |
